# Internet-Based Birth-Cohort Studies: Is This the Future for Epidemiology?

**DOI:** 10.2196/resprot.3873

**Published:** 2015-06-12

**Authors:** Ridvan Firestone, Soo Cheng, Neil Pearce, Jeroen Douwes, Franco Merletti, Costanza Pizzi, Emanuele Pivetta, Franca Rusconi, Lorenzo Richiardi

**Affiliations:** ^1^ Centre for Public Health Research Massey University Wellington New Zealand; ^2^ Department of Medical Statistics, Department of Non-communicable Disease Epidemiology London School of Tropical Medicine London United Kingdom; ^3^ Department of Medical Sciences Cancer Epidemiology Unit University of Turin Turin Italy; ^4^ Unit of Epidemiology Meyer Children’s University Hospital Florence Italy

**Keywords:** Internet, epidemiology, birth cohort

## Abstract

**Background:**

International collaborative cohorts the NINFEA and the ELF studies are mother-child cohorts that use the internet for recruitment and follow-up of their members. The cohorts investigated the association of early life exposures and a wide range of non-communicable diseases.

**Objective:**

The objective is to report the research methodology, with emphasis on the advantages and limitations offered by an Internet-based design. These studies were conducted in Turin, Italy and Wellington, New Zealand.

**Methods:**

The cohorts utilized various online/offline methods to recruit participants. Pregnant women who became aware volunteered, completed an online questionnaire, thus obtaining baseline information.

**Results:**

The NINFEA study has recruited 7003 pregnant women, while the ELF study has recruited 2197 women. The cohorts targeted the whole country, utilizing a range of support processes to reduce the attrition rate of the participants. For the NINFEA and ELF cohorts, online participants were predominantly older (35% and 28.9%, respectively), highly educated (55.6% and 84.9%, respectively), and were in their final trimester of pregnancy (48.5% and 53.6%, respectively).

**Conclusions:**

Internet-based cohort epidemiological studies are feasible, however, it is clear that participants are self-selective samples, as is the case for many birth cohorts. Internet-based cohort studies are potentially cost-effective and novel methodology for conducting long-term epidemiology research. However, from our experience, participants tend to be self-selective. In marked time, if the cohorts are to form part of a larger research program they require further use and exploration to address biases and overcome limitations.

## Introduction

Health research is becoming increasingly complex due to the employment of complex protocols (eg, birth and pregnancy cohort studies); large sample sizes; and novel participant retention strategies [1,2] resulting in increased research costs [3] and low response rates. As a consequence, researchers may need to move beyond traditional methods and explore new and innovative means of conducting valid research more efficiently. Some have argued that health researchers have fallen behind the business world in the use of the Internet [4]. There are different forms of electronic-based methods that can be used for health research, namely, (1) low-technology methods, such as e-mails with appended surveys; (2) electronic bulletin boards, such as an electronic message distribution system set up by independent operators; and (3) the World Wide Web, such as free online research tools. The latter approach requires more comprehensive systems, such as software-specific programs for the research, and it is technically more demanding than low-technology systems [5]. To date, it is clear that using Web-based research methodologies is an emergent trend among a variety of health research disciplines [6-8]. The aim of this paper is to describe 2 Internet-based birth cohort studies as potential models to learn how to develop and conduct, in the future, better Internet-based epidemiological research.

For epidemiological research purposes, utilizing the Internet is currently considered a novel approach. However, its use could become more widespread, at least for longitudinal studies, for the following reasons: (1) the Internet is becoming more accessible and it is globally used[9,10]; (2) in many situations, Web-based research is relatively inexpensive to set up and maintain[5,11,12];(3) the Internet allows for a greater sampling frame for a wider target population, including populations in areas that typically could not be accessed using traditional methods for recruitment [9,12];(4) automated data entry allows the data to be collected in a format suitable for analysis while avoiding data entry errors [5];and (5) combined or individual use of low- and high-technology systems offers a variety of data collection methods that may increase participation rates [13-15].As discussed in recent papers [7,16],baseline selection introduced by recruitment via the Internet may alter the confounding patterns originally present in the source population, but this does not necessarily translate into selection bias in the exposure-outcome estimates obtained in longitudinal studies [17].In this paper, we describe the methodological collaboration between 2 Internet-based birth cohort studies designed to investigate the association between early life exposures and the health of babies through to young adulthood, an international first. The Nascita e INFanzia gli Effetti dell’Ambiente (NINFEA) cohort was established online in Italy in 2005 [7]. From this study, the Early Life Factors (ELF) cohort was designed and implemented online in New Zealand (NZ) in 2008.

The goal for the NINFEA and ELF birth cohorts was to investigate the association between early life factors, early environmental exposures, and noncommunicable diseases. A life-course epidemiology approach was used to investigate exposures at various time points, including the prenatal and early postnatal periods and subsequent postnatal life. This approach assessed the effects of exposures at several stages during the life-course [18], and their interactions, in order to fully understand the causes of a variety of health conditions. For both cohorts, the first 3 phases consisted of similar questionnaires to allow for pooled analyses between the 2 countries. Cohort discrepancies are related to differences in social and cultural aspects relevant to each country and differences in research expertise and interests between the research groups.

## Methods

### Overview

Since recruitment through the Internet is less intensive compared with traditional methods, an advantage of the online approach is that cohorts can recruit for many years. Accordingly, NINFEA is a dynamic cohort with ongoing recruitment and a minimum target of 7,500 participants; however, we report data last downloaded in March 2015. The ELF cohort was a feasibility study and obtained a minimum target of 5,000 participants; recruitment ended at the end of 2012.

### NINFEA Study

The NINFEA cohort started as a pilot study in the city of Turin, Italy, in July 2005 and has been gradually extended to the rest of Italy. The original study protocol and subsequent amendments have been approved by the Ethical Committee of the San Giovanni Battista Hospital—CTO/CRF/Maria Adelaide Hospital, Turin, Italy. Members of the cohort are children of mothers who have access to the Internet, have enough knowledge of the Italian language to complete an online questionnaire, and volunteer to participate at any time during the pregnancy. They register through the project Web site and complete the first questionnaire that lasts approximately 30 minutes. While the Web site has always been public and accessible from any part of Italy (and the world), the methods for advertisement of the existence of the study have changed over time.

All women participate online, although NINFEA is advertised using both offline and online methods. Offline methods involve the collaboration of health personnel and, therefore, target a prespecified catchment population. Currently, the NINFEA study is actively advertised in the city of Turin, in the Tuscany Region and, with a lower intensity, in the Piedmont Region (of which, the city of Turin is the capital). In these areas, leaflets and posters were distributed, and the study was introduced to pregnant women when they attended hospitals or family clinics for reasons related to their pregnancy. Online recruitment includes recruitment through the Internet (eg, Web sites, forums, social networks) and the media.Until March 2015, approximately 16% of participants were recruited via a passive mode, 82% were recruited actively, and 2% of participants comprised both modes. A total of 7003 pregnant women were recruited in the study as of March 2015. The 3 most represented Italian Regions in the NINFEA cohort are the Piedmont Region (62% of the participants), the Tuscany Region (22%), and the Lombardy Region (4%), while the most represented municipality is Torino (45%). About one-third of the participants are from central urban areas, almost 50% from peripheral urban areas, and the remaining 20% are from rural areas.

### ELF Study

The primary location for the ELF cohort is Wellington, New Zealand, but additional study sites are located in the other main city centers (eg, Christchurch and Auckland). Ethical approval was obtained in 2007 from Massey University, New Zealand (MUHEC Application 07/62). Pregnant women who were 16-years-or older were eligible to participate in the study. The ELF cohort recruited pregnant women at “parent and child shows” located in the main urban centers. Parent-child shows are large-scale events, marketed at expecting and experienced parents. People pay a small fee to enter these shows because it is a “1 stop shop” destination to purchase standard and newly available products (eg, food), services (eg, child-care), recreation and education programs (eg, developmental courses), and specialist advice (eg, child psychologist). The shows are attended by more than 22,000 people annually. The ELF study used other recruitment avenues including: information inserted in antenatal care booklets, promotional posters in hospitals and sonography clinics, and participants who enrolled through an Internet search engine. Thus, the study population included any expecting mothers, new and experienced, recruited through parent-child shows and other avenues, with access to the Internet. Participants were offered a “postal” option (offline) if they did not have access to the Internet, or if they preferred the offline option.

A final total of 2197 women were recruited in the study from September 2008 to September 2012. A large proportion of the participants were from Wellington (43.5%) with the other participants from Auckland (37.5%) and Canterbury (11.8%). A small proportion was from other regions (7.2%); and for less than 1% we had no current address. From the 2197 pregnant women recruited, 1,155 (52%) were categorized as lost to follow-up. The reasons were: (1) attrition to follow-up (81%); (2) participants who later declined to take part (12%); (3) missing information (1%); and other reasons (4%) such as miscarriage, nonviable pregnancy or death of the baby, and moved to another country and subsequently withdrew from the study. The final study sample analyzed in this paper is 1,042 participants. The majority of respondents (55%) took part via an offline mode, compared to 44.9% of online participants, and most women were recruited from the parent-child shows (73.2%), as described earlier.

### Follow-Up Measures

#### NINFEA Study

When it is time to complete a follow-up questionnaire, participants are invited to access the Web site using their username and password. The follow-up questionnaire remains accessible for a number of months after the first invitation, while women are reminded of the questionnaire via e-mail, telephone calls, short message service (SMS) texts, and regular mail. For example, the 6-month questionnaire can be completed until the child turns 15 months old; after that, the questionnaire is closed and the woman is considered as “lost to follow-up.” Based on this definition, the attrition proportions for each of the follow-up questionnaires were estimated on the NINFEA database version 15.03. Out of all pregnant women recruited at baseline, 88% completed the 6-month questionnaire, 83% completed the 18-month questionnaire, and 78% completed the 4-year questionnaire. These proportions refer to the overall participation, including, for example, miscarriages and stillbirths in the denominator.

#### ELF Study

As described earlier, in an attempt to reduce the attrition rate, we sent out quarterly reminders and newsletters and made the follow-up online questionnaires available for an indefinite period. Based on the ELF database version 13.08, out of all the pregnant women recruited at baseline, 47.4% completed the Phase I questionnaire and 52.5% participants were identified as lost to follow-up, as defined by the proportion of participants that did not submit the questionnaire after at least 3 follow-up reminders in Phase I of the study. Of those that participated, the participation of onliners (44.9%) as compared to offliners (55%) was proportionately less. A specific focus on recruiting only online participants may have reduced the attrition rate of the ELF cohort.

### Cohort Measures

#### NINFEA Study: Questionnaires

The cohort is multipurpose and collects information on a broad range of exposures and outcomes. NINFEA involves 3 main questionnaires and subsequent short questionnaires targeting specific outcomes and/or exposures. Further follow-up questionnaires will be added in the future. Table 1 summarizes the domains that are currently investigated in the NINFEA study. Further information is available on the inventory of European birth cohorts.

After the first baseline questionnaire (completed during pregnancy), participants complete 2 other main (30 minutes long) online questionnaires at 6 months and 18 months after delivery. Long-term follow-up continues with short online questionnaires focusing on specific outcomes and linkage with health-related databases (eg, inpatient registry, prescription registry, etc).

When it is time to complete a follow-up questionnaire, mothers are contacted by e-mail asking them to access the Web site and complete the questionnaire. Nonresponders are additionally contacted first by e-mail and then by telephone, SMS texts, and regular mail. Contact between participants and the research team is also maintained using the NINFEA Facebook page, which is updated weekly.

#### NINFEA Study: Biological samples

The NINFEA study also involves collection of saliva samples from the mothers and the children, which commenced in 2009. At the time of the Phase II questionnaire, when the child is aged 6 months, women are asked if they want to participate in this part of the study. Upon acceptance, they receive 2 self-collection kits, 1 for the mother and 1 for the child. Saliva is then stored at -80°C mainly for extraction of DNA to be used in genetics and epigenetic-based studies. To involve the complete cohort, participants who took part previous to the implementation of the biological study were invited to participate in the donation of saliva samples at the Phase III and IV questionnaire stages. As of February 4, 2015, a total of 2,864 mother-child pair saliva samples have been collected.

#### ELF Study: Questionnaires

Modeling the work from the NINFEA study, the ELF study is also multipurpose and aims to examine a wide range of exposure information collected at important milestone time points, starting at the prenatal stage. Based on our interest in early life exposures, the participants were also asked to report whether they were ever diagnosed with a wide range of medical conditions, including asthma, allergies, high blood pressure, heart conditions, diabetes, stroke, thyroid problems, psychological problems, sexually transmitted infections, diseases of the reproductive system, and more. Table 1 lists each questionnaire and details the information collected at each phase.

Following the completion of the Phase I questionnaire (during pregnancy), regular reminders about the study were e-mailed and postal-mailed to each individual every 3 months. In addition, a quarterly newsletter was sent to all participants to provide an update on the study, and it served as a reminder to renew participant contact details for follow-up purposes. The study Web site includes an electronic inquiry, with a toll-free telephone number that helps participants to maintain contact with the research team.

Presently, ELF includes a short questionnaire on birthing, developmental milestones, sleep patterns, environmental exposures, and respiratory health. Follow-up questionnaires occur at 3 months, 15 months and at 2 years of age. For follow-up, mothers were contacted by e-mail, asking them to access the Web site and complete the questionnaire. Additionally, any nonresponders were contacted first by e-mail and then by telephone and regular mail.

**Table 1:** Schedule of questionnaire phases, by Internet cohort.

Questionnaire Phase	Cohort Schedule	Internet Cohort	
		ELF	NINFEA
Phase I	ELF: Prenatal	Social and demographic characteristics	Social and demographic characteristics
	NINFEA: Prenatal	Current and historical occupational exposures	Current and historical occupational exposures
		Domestic environmental exposures	Domestic environmental exposures
		Medical history	Medical history
		Medication use and duration	Medication use and duration
		Reproductive and pregnancy history	Reproductive and pregnancy history
		Maternal weight and diet	Maternal weight and diet
		Lifestyle behaviors	Lifestyle behaviors
		Fitness and physical activity	Fitness and physical activity
		Sleep habits	N/A
		Access to the study	Access to the study
		N/A	Selected information about the partner
Phase II	ELF: 3 mo	Birth outcomes and neonatal tests	Birth outcomes
	NINFEA: 6 mo	Infant anthropometric measures	Infant anthropometric measures
		Infant health	Infant health
		Feeding practices and related behaviors	Feeding practices and related behaviors
		Infant sleep habits	Infant sleep habits
		Contact with other children	Contact with other children
		Domestic environment	Domestic environment
		Work and farming environment	N/A
		Maternal lifestyle factors	Maternal lifestyle factors
		N/A	Update of the baseline questionnaire
Phase III	ELF: 15 mo	Infant anthropometric measures	Infant anthropometric measures
	NINFEA: 18 mo	Feeding practices	Feeding practices
		Health and well-being of the mother	Health and well-being of the mother
		Health and well-being of the infant	Health and well-being of the infant
		Sleep patterns	Sleep patterns
		Contact with other children	Contact with other children
		Domestic environment	Domestic environment
		Farming/animal exposures	N/A
		Work/occupational exposures	Work/occupational exposures
		Smoking	Smoking
		Leisure activities	Leisure activities
		Bonding between child and parent	Bonding between child and parent
Phase IV	ELF: 2 y	Food frequency over a 4- week period	Anthropometric measures
	NINFEA: 4 y	Food habits	Anthropometric/Cognitive development
		Oral health	
		Physical activity	
		Respiratory health	
Phase V	ELF: N/A	N/A	Anthropometric/Respiratory health
	NINFEA: 7 y		

## Results

### Subject Characteristics

#### NINFEA Study

Selected characteristics of the NINFEA study participants are reported in Table 2. Women clearly self-selected; that is, the majority of participants was aged at least 32 years, had a university degree, and most were experiencing their first pregnancy [16]. However, there was still heterogeneity in most of the exposures of interest, as shown by 8% who smoked and 34% who drank alcohol during the first trimester of pregnancy in the cohort.

#### ELF Study

Selected characteristics of the study participants of the ELF study are reported in Table 3, stratified by method of participation (online or offline). The majority of women were aged between 26-35 years, with an average age of 31 years across both groups. Overall, a large number of women (74%) self-identified their ethnicity as being NZ European, with the remainder identifying either as Māori (indigenous people of NZ; 9.8%) or other (15.9%); while less than 1% did not state their ethnicity. More than half of the participants (54.9%) had 1 or more pregnancies prior to the current pregnancy, and about 57% participated in the study while in their third trimester. Across both groups, 82.8% of the participants had a tertiary level qualification, and an overall 45% earned more than the highest median weekly income in NZ (total average from all sources: $550 per week) [20]. The postal codes and the New Zealand Deprivation Index 2006 (NZDep2006) were used to create a standardized measure of socioeconomic deprivation. Based on the 2006 New Zealand Census, the index combines 9 census variables. The index provides a deprivation score for each small area unit (“meshblock”) in NZ. These meshblocks are defined by Statistics New Zealand as geographical units, which contain a median of 90 people. Each meshblock is categorized between 1 (least deprived) and 10 (most deprived) [19]. For our analyses, deciles were grouped into quintiles: 1-2 (least deprived); 3-4; 5-6; 7-8; 9-10 (most deprived). Approximately 50% of the participants were from the least deprived socioeconomic position.

Currently, the ELF cohort data has been used to conduct quality checks and descriptive analyses, including the comparison of online versus offline participants (Table 4). However, the notable finding from this table indicates a significant difference in women who reported that they had “ever” smoked during pregnancy compared to those women who reported “never” smoking during pregnancy (*P=* .002). Additionally, out of all the women who answered the question about quitting smoking (n=1,019), women were significantly more likely to report “smoked but quit” than to report “no smoking” during pregnancy (*P=* .01).

**Table 2:** Selected characteristics of participants in the NINFEA study^a^.

Characteristics		N=7003 (%)
Maternal age, y		
	≤25	5.0
	26-31	33.3
	32-35	35.0
	≥36	26.7
	Missing data	0.1
Maternal origin		
	Italian born	95.8
	Non-Italian born	4.2
Maternal residence		
	North Italy	71.3
	Central Italy	25.2
	South Italy	3.5
Maternal educational level		
	Primary school	5.8
	Secondary/college	33.8
	University/tertiary	58.6
	Missing data	1.8
First pregnancy		
	No	32.7
	Yes	63.3
	Missing data	4.0
Stage of pregnancy at recruitment		
	Trimester 1	15.3
	Trimester 2	36.0
	Trimester 3	48.5
	Missing data	0.2
Smoking during pregnancy		
	No	89.3
	Yes	8.4
	Missing data	2.4
Drinking during pregnancy		
	No	64.4
	Yes	33.7
	Missing data	1.9

^a^Database version 15.03 (March 2015).

**Table 3:** Selected characteristics of participants in the ELF cohort.

Characteristics		Online	Offline	All	*P* values^c^
		n=468 (%)	n=574 (%)	N=1,042 ($)	
Maternal age, y	≤25	12.6	15.9	14.4	
	26-31	37.5	34.4	35.8	
	32-35	28.9	31.5	30.3	
	≥36	21.0	18.2	19.4	
	Missing data	n=1	n=3	n=4	*P=* .24
Partner status	No	3.9	5.8	4.9	
	Yes	96.2	94.2	95.1	
	Missing data	n=0	n=1	n=1	*P=* .15
Ethnicity	NZ European	78.6	70.8	74.3	
	Maori^a^	7.9	11.4	9.8	
	Other	13.5	17.8	15.9	
	Missing data	n=0	n=2	n=2	*P=* .01
Regions participating	Auckland	33.6	40.6	37.5	
	Wellington	45.8	41.6	43.5	
	Canterbury	13.1	10.8	11.8	
	Other	7.5	7.0	7.2	
	Missing data	n=3	n=0	n=3	*P=* .12
NZDep06^b^	Quintile 1	26.7	26.5	26.6	
	Quintile 2	25.2	22.3	23.6	
	Quintile 3	22.0	22.0	22.0	
	Quintile 4	16.0	17.7	16.9	
	Quintile 5	10.1	11.5	10.9	
	Missing data	n=4	n=0	n=4	*P=* .75
Highest educational level	Primary school	0.0	0.5	0.3	
	Secondary/college	15.1	18.5	16.9	
	University/tertiary	84.9	81.0	82.8	
	Missing data	n=3	n=5	n=8	*P=* .09
Total household income	$1-$40,000	6.4	10.6	8.7	
	$40,001-$70,000	22.9	21.5	22.1	
	$70,001-$100,000	23.6	24.4	24.0	
	$100,001+	47.0	43.5	45.1	
	Missing data	n=45	n=57	n=102	*P=* .12
First pregnancy	No	56.7	53.3	54.9	
	Yes	43.3	46.7	45.1	
	Missing data	n=1	n=2	n=3	*P=* .27
Stage of pregnancy	Trimester 1	5.3	1.0	3.0	
	Trimester 2	31.0	16.2	22.8	
	Trimester 3	53.6	59.8	57.0	
	Full term	10.1	23.0	17.2	
	Missing data	n=0	n=0	n=0	*P*<.001

^a^Maori = Indigenous people of New Zealand.

^b^NZDep06 Quintiles = New Zealand Deprivation Index 2006: a scale based on Census information, where 1 represents 10% of the least deprived and 10 represent 10% of the most deprived people in New Zealand.

^c^ *P* values = chi-square test

**Table 4:** Selected key exposures of online versus offline participants of the ELF cohort.

Characteristics			Online	Offline	All	*P* value
			n=468 (%)	n=574 (%)	N=1,042 (%)	
BMI (kg/m^2^)						
	Prepregnancy	BMI<18.5	3.3	3.1	3.2	
		BMI 18.5-<25.0	50.5	57.5	54.3	
		BMI 25.0-<30.0	31.1	25.4	28.0	
		BMI≥30.0	15.1	14.0	14.5	
		Missing data^d^	n=11	n=23	n=34	*P=* .14
Smoking	During Pregnancy	No	94.4	89.0	91.4	
		Yes	5.6	11.0	8.6	
		Missing data	n=0	n=1	n=1	*P=* .002
Quit Smoking	During Pregnancy	Smoked but Quit	95.5	91.7	93.4	
		No Smoking	4.5	8.3	6.6	
		No. of participants reporting	n=463	n=556	n=1,019	*P=* .01
Alcohol	During Pregnancy	No	75.5	67.2	70.9	
		Yes	24.5	32.8	29.1	
		Missing data	n=6	n=0	n=6	*P=*.004
Drugs^a^	During Pregnancy	No	97.4	96.7	97.0	
		Yes	2.6	3.3	3.0	
		Missing data	n=3	n=0	n=3	*P=* .49
Comorbidities^b^ (ever vs never)	During Pregnancy	Respiratory Diseases	42.6	43.7	43.2	
		Missing data	n=1	n=0	n=1	*P=* .71
		STI^c^	18.7	21.0	20.0	
		Missing	n=2	n=3	n=5	*P=* .34
		Mental Health Disorders	31.6	29.0	20.0	
		Missing data	n=2	n=2	n=4	*P=* .37
		Reproductive Diseases	38.4	32.8	35.3	
		Missing data	n=2	n=3	n=5	*P=* .058
		High blood pressure	6.2	6.6	6.5	
		Missing data	n=2	n=3	n=5	*P=* .79
		Diabetes	1.1	0.9	1.0	
		Missing data	n=4	n=0	n=4	*P=* .83
		Other	83.7	81.9	82.7	
		Missing data	n=1	n=0	n=1	*P=* .43

^a^Illicit use during pregnancy.

^b^Comorbidities=numbers do not add up to 100 due to coexisting morbidities.

^c^STI=Sexually transmitted infections.

^d^Missing data was not included in the analyses.

## Discussion

### Principal Findings

The NINFEA and ELF studies are Internet-based cohorts examining protective and risk factors for a range of noncommunicable diseases in young children. These online birth cohort studies are the first of their kind in Italy and NZ. Both cohorts’ participants differed in age structure, with the largest group in the 32- to 35-year-old age group (35.7%) for NINFEA. The ELF cohort’s maternal age was predominantly younger (26- to 31-year-old age group). These age groups typically represent the median maternal age for both countries (NZ: 29 years; Italy: 31.3 years) [21,22], which explains much of the changes in the reproductive process (ie, birth delay) and stabilized fertility rates since the 1960s and 1970s [22. A comparison between the NINFEA cohort and the general population revealed that NINFEA participants are mothers with lower parity, higher education level, and lower frequency of smoking during pregnancy [16]. For the ELF cohort, online participants were notable by ethnicity and stage of pregnancy (trimester 3 having the highest participation for both online and offline). The latter characteristic falls in line with the NINFEA’s previous analyses, where women in their first trimester of pregnancy have a lower proportion of completed items from the baseline questionnaire [16]. Indeed, these findings are characteristic of Internet-based cohorts being a more self-selective sample of their respective source populations, and the timing (or in this case, the stage of pregnancy) for inviting participants to be take part in an online survey is an important consideration in order to attain complete responses and better respondent rates. Key risk factors of both the NINFEA and ELF cohorts indicated a reasonable comparability of participants who were smokers (8.4% and 8.6%, respectively) and drank alcohol (33% and 29%, respectively) during pregnancy, with clear differences between online and offline users in the ELF cohort. As more health outcomes data becomes available for the ELF cohort (ie, completion of subsequent phases), further analyses—including early life growth trajectory pathways to several health outcomes—will provide meaningful and useful interpretation.

The preliminary findings presented here show that an Internet-based cohort is feasible. Our investigation also highlights 3 major strengths that support the notion that Internet-based cohort studies are feasible and may have advantages in comparison to traditional cohorts: (1) given the prospective longitudinal nature of a cohort study design, an Internet-based approach can provide a significant research resource, particularly in the potential for expanding the breadth of a sampling frame and automated data downloading and cleaning that reduces the costs for administering a project long term; (2) the mode of Internet participation (eg, online questionnaires) has the potential to include multiple geographical sites for a long duration of time and to include large numbers of participants in the study (since recruitment through the Internet is less intensive as compared with traditional methods, an advantage of the Internet approach is that cohorts could recruit for many years; furthermore, there are provisions for identical cohorts to be established in other countries, and the online nature of the study could preclude additional costs for participation of mothers at an international level); and (3) as the protocols and online questionnaires are comparable for some phases of the ELF and NINFEA cohorts, particularly on future analyses on specific exposures and outcomes, this will allow for subsequent pooled analyses (these are currently being planned as the follow-up for each phase becomes more complete and for later phases when the children are of school age).

Limited access to the Internet, particularly for participants from a lower socio-economic background, may result in a selective cohort. Although this selection is not likely to introduce problems of validity in the associations measured, there may be issues of limited exposure heterogeneity in the study subjects. This would happen when the exposure of interest is strongly associated with participation and that there is limited variability in the exposure to investigate its effect on the outcome of interest. However, this problem is likely to be limited as in many countries, including Italy and NZ, the majority of the population not only has access to the Internet, but also access the Internet from a handheld mobile device such as a smartphone or an iPad (Italy: 58%; NZ: 88% in the whole population in 2012 and 2013 [23,24]). In addition, for some exposures, baseline selection may actually increase heterogeneities. For example, if high maternal age were the exposure of interest, having 25% of the cohort aged at least 36 years at delivery would increase the statistical efficiency of the cohort. Moreover, it is important to note that due to the Internet-based design and source population of the NINFEA and ELF cohorts, restricting the source population (like in our cohorts) are more likely to reduce issues of internal validity. This issue has been recently discussed the general consensus is that “representativeness” will depend on the context of a particular study, and thus it is a secondary issue [25]. Other researchers suggest that representativeness should be avoided, particularly if the study design incorporates an intentional nonrepresentative sample for practical reasons (eg, restricting the study to specific participants); minimizing bias by comparing subgroups; and if the focus was on 1 or more population subgroups [26]. This is the case for the cohorts currently presented in this paper, thus restricting the source population and internal analyses should not introduce serious issues of bias.

However, an important characteristic in all birth cohort studies where participants are followed-up is “attrition,” and we acknowledge that this is a particular issue for the ELF cohort. Anecdotally, ELF participants provided comments on addressing attrition or lost to follow-up. This included ideas of shorter questionnaires, reducing the interval time for the data collection phases, setting up electronic diary reminders with the participants, being very clear with participants to utilize the offline option if they are “not Internet-savvy,” and simplifying the Internet processes for enrollment (ie, there were some technical glitches that prevented participants from registering in a timely manner). These are important learnings from participants’ perspectives, and the authors accept that the points highlighted here should be considered for any future Internet-based research. Moreover, there is emerging work examining the follow-up of Internet-based epidemiological studies, and the findings advocate using an offline enrollment campaign as a potentially useful aid to achieve higher participation and to limit lost to follow-up. Based on the NINFEA and ELF cohort experiences, we cannot conclude whether attrition is higher or lower in Internet-based cohorts than in traditional cohort studies. As further phases are completed and the cohorts experiment with different online mechanisms (eg, use of social media tools), this issue will become clearer and will produce potential strategies to alleviate attrition at follow-up when using the Internet as a primary method of recruitment and data collection.

### Completed and Ongoing Work and Future Directions

The initial work focused on the use of the Internet to conduct cohort studies.

First, some studies demonstrated empirically that baseline selection (or restriction) in cohort studies does not result in biased associations [16]. This has previously been recognized [27], and further support in the context of Internet-based research is needed. Data from the NINFEA study and the population-based birth registry of the Piedmont Region, Italy, were used to show that the confounding pattern in the NINFEA cohort differs from that of the general population, but this difference is not necessarily associated with a stronger overall confounding effect [16]. Simulation studies in which both the exposure of interest and an unmeasured strong risk factor for the outcome of interest, assumed to be independent in the general population, are strong determinants of the probability of participating in the Internet-based cohort were also performed, showing that even in the worst-case scenario, the magnitude of the bias introduced was small [16].

Further work to evaluate methods of recruitment for an Internet-based cohort and their potential effects on the study validity is ongoing. For example, the efficiency of a pilot advertisement campaign in Facebook, estimating a cost of €20 per participant, has been recently studied [30]. In addition, we found that both in the NINFEA and in the ELF cohort, the source of information (offline vs online) was associated with attrition at follow-up.

Studies on specific outcomes are also ongoing, in particular on growth in the first years of life and on wheezing. Data from the NINFEA cohort and 2 other non-Internet-based cohorts have been used to compare different approaches to model growth in the first 4 years of life [28], the association between several maternal prenatal exposures and weight trajectories in infancy were examined [17,28], and the paper highlighted a range of modeling options to estimate salient features of growth in weight in infancy and early childhood. However, the most useful was the SITAR (super-imposition by translation and rotation) model because of its flexible and pragmatic approach for life-course epidemiology inquiries. Finally, the NINFEA cohort participates in several collaborative studies among European cohorts, including those conducted under the CHICOS coordination project [29-34].

The 2 Internet-based cohorts presented in this paper had similar participant characteristics despite the differences in methods, data collection time frames, and source populations. Internet-based recruitment for epidemiological studies has the potential to expand a broader geographical coverage. However, online recruitment could introduce difficulties, particularly in the collection of biological samples, and it limits the capability to take standardized measurements (eg, weight, height). The NINFEA cohort protocol includes collection of saliva samples when children turn 6 months old, but it does not include cord or maternal blood sampling. There is, however, the potential of nesting ad-hoc studies in a subsample of the cohort to mitigate this issue.

### Conclusions

There is much to learn about how to include the Internet as a valuable tool in epidemiological research. Over time, technological advances can only further aid in overcoming much of the current shortcomings, particularly in increasing follow-up and reducing the attrition rate. We encourage future studies to incorporate the Internet more strategically to decrease the limitations of individual and population-based approaches in epidemiological study designs.
